# Determinants of effective organisational capacity training: lessons from a training programme on health workforce development with participants from three African countries

**DOI:** 10.1186/s12889-019-7883-x

**Published:** 2019-11-26

**Authors:** Woldekidan Kifle Amde, Bruno Marchal, David Sanders, Uta Lehmann

**Affiliations:** 1 School of Public Health, University of the Western Cape, Cape Town, South Africa; 2 Department of Public Health, Institute of Tropical Medicine, Antwerp, Belgium

**Keywords:** Alignment, Capacity development, Context, Blended learning, Health workforce development, Motivation, Organisational capacity development, Retention, South-south cooperation, Training

## Abstract

**Background:**

Health systems in sub-Saharan Africa face multifaceted capacity challenges to fulfil their mandates of service provision and governance of their resources. Four academic institutions in Africa implemented a World Health Organisation-funded collaborative project encompassing training, curriculum development, and partnership to strengthen national leadership and training capacity for health workforce development. This paper looks into the training component of the project, a blended Masters programme in public health that sought to improve the capacity of personnel involved in teaching or management/development of human resources for health. The paper aims to explore factors influencing contribution of training to organisational capacity development.

**Methods:**

We chose a case study design. Semi-structured interviews were held with 18 trainees that were enrolled in the training programme, and who were affiliated to health ministries or public health training institutions. We gathered additional data through document reviews, observation, and interviews with 14 key informants associated with the programme and/or working in the collaborating institutions. The evidence gathered were analysed thematically.

**Results:**

Thirteen of the 18 training participants stayed in the target institutions and contributed to improved capacity of their institutions in the fields of management, policy, planning, research, training, or curriculum development. Five left for private and international agencies due to dissatisfaction with payment, work conditions, or career prospect.

Factors that were associated with the training, trainees, and the institutional and broader context, determine contribution of training to organisational capacity development. These include relevance of newly acquired knowledge and skills set of trainees to the role/position they assume in the organisation; recognition of trainees by employing organisations in terms of promotion or assignment of challenging tasks; and motivation and retention of trained staff.

**Conclusion:**

Training, even if relevant and applicable, makes no more than a ‘latent’ contribution, one which is activated and realised through alignment of clusters of interacting contextual and relational factors related to the target institutions and trained personnel. While not predictable, implementers need to focus more deliberately on the likely interaction and best possible alignments between training relevance, student selection for potential to contribute, recognition and career advancement potential.

## Background

Health systems in sub-Saharan Africa face multifaceted capacity challenges to fulfil their mandates of service provision and governance of their resources. Wide-ranging capacity development interventions exist to address these limitations. Many countries in Africa experience acute staff shortages and poor performance of the health workforce. This is related to inadequate attention given to workforce development from institutions, which either train or employ human resource managers [1]. The lack of leadership and management across different levels of the health sector is often manifested in the practice of appointing clinical staff to human resource management (HRM) roles [2–4] without the required training to execute HRM functions across policy, leadership, education, partnership and finance spectrums [5, 6].

A number of human resource management interventions in low-and middle-income countries have focused on training as a principal strategy for improving health workers’ performance and boosting capacity, mostly among frontline providers [7, 8]. Similar trends also prevail in other capacity development initiatives in the health sector. A study mapping a wide range of interventions to improve national and institutional capacity in health research in Africa found that ‘many efforts are geared towards individual capacity building, with indirect benefit to institutions’ [8].

**Fig. 1:**
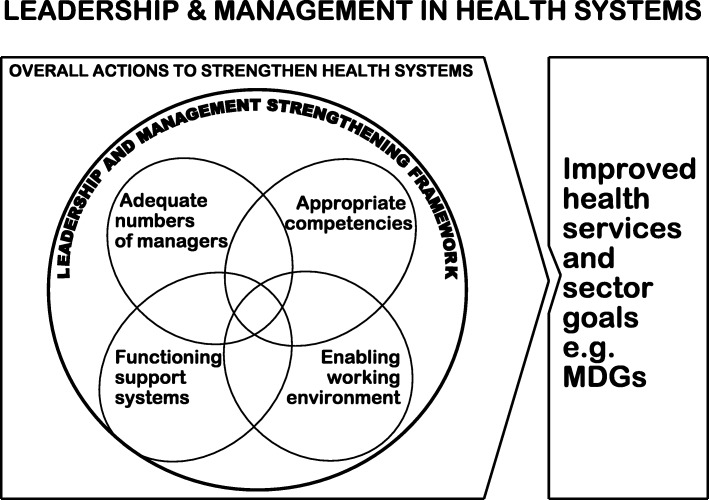
WHO’s leadership and development framework

Scholars have noted and criticised the disproportionate focus on building individual level capacity as a primary strategy to the neglect of other systems level issues [9–12]. The criticisms highlight the links between individual and organisational level capacity development, which are often taken for granted [13]. A World Bank evaluation (2008) that explored whether training results in organisational capacity development identified the elements that need to be in place for training to effect the desired transformations: quality training, and an enabling work environment [14]. Other authors point out the importance of the attributes of trainees and of having an appropriate curriculum [15, 16]. All these dimensions are captured in the WHO’s leadership and development framework (Fig. 1) which emphasises the dynamic interaction between numbers, competencies, support systems and working environments [17].

The capacity development literature, too, emphasises the need for a systems perspective that looks into interactions between the different capacity levels (individual, organisational, or environmental), and investigates the issues that nurture or undermine capacity development at each level [9, 18–24]. As Hongoro and Normand point out: ‘Organizational and system arrangements define the incentive context for health workers and influence both organizational and individual performance’ [25]. Similar calls for a systems approach have also been made in the context of interventions and research to develop public health and research capacity [7, 8, 25–29]. According to Bennet, “Human resource management systems affect workers’ capability and their perception of that capability, through such mechanisms as training, supervision, and more concrete incentives such as remuneration, promotion, and performance review processes” [29].

With respect to engagement post training, literature further establishes the importance of recognising and supporting individuals that are tasked with the responsibility of spearheading change, and identifies seniority as one of the attributes these individuals need to possess [30–32], hence imperative to put great care in the selection of these individuals.

With a focus on the use of training on health workforce development as a strategy to leverage organisational capacity development, both in health ministries and public health training universities, this paper explored the factors that influence contribution of training to organisational capacity development.

The paper is part of a broader research project that focuses on examining a collaboration among four academic institutions in Africa (year 2009–2015), to strengthen leadership and training capacity for health workforce development at both national and regional levels. The collaboration arose in response to a call by – and financial support from – the World Health Organisation [33]. The collaboration has three dimensions: building a regional network, curriculum/programme development in partner institutions, and training of critical mass of experts in the field of health workforce development. The paper looks at one component of the collaboration, a blended Masters in Public Health programme with a focus on Health Workforce Development. The programme was offered in the University of the Western Cape, South Africa, through a blended approach, a combination of distance learning and short face-to-face contact. The programme consists of six HRM-focused and public health modules, and a thesis on an issue focused on human resources for health (HRH) in their country.

## Methods

We adopted a qualitative case study research design due to its suitability for investigating ‘why’ and ‘how’ questions [34–36]. Training participants enrolled in the training programme and affiliated to target institutions across the three countries are the focus of the paper. Eighteen participants (6 each from Mozambique, Rwanda, and Ethiopia) underwent the training after being nominated to take part in the programme by their home institutions: health ministries or universities. Sixteen trainees were located, and assumed leadership or trainer roles where human resource management was one of the central foci. This proximity to HRM-focused roles/functions had been a criterion for their nomination to the training programme. Additional criteria used in the selection of trainees include academic qualifications, and local considerations of service duration and representation of sub-national groups [33].

Semi-structured in-depth interviews [37–39] were held with all the 18 training participants. We gathered additional data through review of documents (project proposals, project agreements, progress reports and correspondence) and observation. Additional interviews were conducted with 14 key informants that were selected purposively on the basis of their proximity to the training programme or participants [38, 40, 41]. They include trainers, mentors, coordinators affiliated with public health training institutions- University of Rwanda, Addis Ababa University in Ethiopia and Mozambique’s Eduardo Mondlane University; and representatives of project implementation partners- University of the Western Cape, South Africa, and the World Health Organisation.

The interviews were held between June 2014 and March 2015. All the trainees had completed the programme during the time of the interview, and all but three had graduated. The interviews were audio recorded with the permission of participants.

Information about prior training and professional background of participants and demographic details were also gathered. The interviews explored perceptions and experiences of actors over a range of matters related to the training programme, their work settings and responsibilities, and factors that enabled or constrained learning or application of acquired competencies in the programme. Probing was an important strategy used to establish adequate understanding [37, 39].

Good rapport with participants was established over the course of the training programme. This was evident in the way participants were willing to be part of the research and the openness they exhibited during the interviews. Field notes [38] were prepared while on the field.

The collected data were analysed thematically [42, 43]. All interviews were transcribed word for word. The transcription was open coded manually with a focus on describing the diversity in the data. The codes were then grouped into more analytical categories. Themes were generated both inductively from the data and deductively from the research question and the literature on capacity development and the systems and complexity approach [38]. Causal loop diagram was used to visualise and communicate complex system interactions [44–46] taking into consideration feedback mechanisms, both enablers and constraints.

Rigour and trustworthiness of the research was ensured through triangulation by seeking convergence of data from multiple data sources and methods [38, 39, 43, 47, 48]. Strategies used to improve rigour and trustworthiness include engagement with participants and context prior to data collection; voluntary participation of respondents; counter checking responses; and soliciting peer feedback [38, 48–50].

Reflexivity was an important tool in this research. Three of the authors were part of the programme intervention either in the design, implementation, monitoring or documentation of the programme. Hence, we acknowledge drawing on and incorporating our experiences.

## Results

### Profile of training participants

Thirteen of the training participants were male and five were female. The median age of participants was 37, and their ages range between 25 and 56 years. With respect to institutional affiliations, 13 were based in health ministries, and five were located in public health training institutions. Looking at the educational background of the participants, half had clinical background and the other half had social science background (Psychology, Education, Sociology, and Administration). At the time of the study, of the 18 training participants, five left their home institutions to private or international agencies due to dissatisfaction about remuneration, work condition and career prospect. Detail information on career progression and capacity application experiences of training participants are presented in Table 1.

**Table 1:** Career progression and capacity application experiences of training participants

Code	Background	Institutional affiliation	Positions – before training	Position – during/after training	Opportunities to apply	Reason for leaving/staying
P2	Social Science	Locally based training institute	Coordinator of the university’s research project site	Coordinator of the university’s research project site	Had few opportunities to apply capacity in current role	Stayed despite lack of recognition by employer and lack of opportunities due to lack of better alternatives, has a non-medical background
P8	Medical Science	Locally based training institute	MD and lecturer	Head of national human resources department	Multiple opportunity to apply capacity within the institution and represent institution in partnership with other institutions	Left despite high recognition by employer, seniority and multiple opportunity to apply capacity, due to career change, lack of commitment to organisation and family commitments
P3	Medical Science	MOH	Hospital director	Head of national human resources department, and later head of the national health department	Multiple opportunity to apply capacity within the institution and represent institution in partnership with other institutions	Stayed despite poor wages due to high recognition by employer, promotion, challenging assignments, and commitment to organisation and service
P14	Medical Science	MOH	Head of human resource at provincial health department	Works for an international agency, advisor to the provincial health department	Former role offers multiple opportunity to practice, current role also offers opportunities for application	Left despite recognition, and challenging responsibilities, due to low salary, possibility to continue working with MOH as a member of external development partner
P15	Social Science	MOH	Human resource officer at provincial health department	Head of human resource at provincial health department	Multiple opportunities to apply capacity within the institution and represent institution in partnership with other institutions	Stayed due to (albeit delayed) recognition by employer, promotion, challenging assignments
P16	Management	MOH	Training officer at MOH	Head of HR of a public hospital	Had opportunity to apply capacity in current and former role	Stayed due to recognition by employer, promotion, challenging assignments
P35	Medical Science	Locally based training institute	Lecturer	Lecturer, teaching HRH–related modules	Multiple opportunities to apply capacity within the institution and represent institution in partnership with other institutions	Stayed due to high recognition by employer, challenging assignments, career advancement, additional income
P36	Social Science	MOH	Procurement manager of a hospital	Head of HR development for a private company	Former role offers little opportunities to practice, multiple opportunity for practice in current role	Left due to low-salary, poor recognition and career prospects
P37	Social Science	MOH	Trainer at national human resource Directorate	Head of HR planning, National HRH Directorate, MOH	Multiple opportunities to apply capacity within the institution and represent institution in partnership with other institutions	Stayed due to recognition, promotion, job satisfaction
P38	Social Science	MOH	Financial Manager, sub-national structure, MOH	Head of HR, public hospital	Had an opportunity to apply capacity in current and former role	Stayed due to recognition, and relevant assignments
P39	Medical Science	Locally based training institute	Researcher and lecturer	Works for an international agency	Former role offers little opportunity to practice, multiple opportunity for practice in current role	Left due to low salary, low career prospects
P40	Social Science	MOH	Director of Health Training Centre in district	Permanent secretary of government in a district, Director General of a district	Had opportunity to apply capacity in current and former role	Stayed due to recognition, promotion
P54	Social Science	Locally based training institute	University research project staff	Lecturer, teaches HR-related courses. Coordinator of academic programmes, project manager	Multiple opportunities to apply within the institution and represent institution in partnership with other institutions	Stayed due to employer recognition, promotion, job security, advanced from project to permanent academic staff
P55	Medical Science	Local-based training institute	University project staff	Lecturer of HRH-related modules, coordinator of HRH-related postgraduate programme	Multiple within the institution and represent institution in partnership with other institutions	Stayed due to recognition; promotion, job security, transitioned from project to permanent academic staff
P56	Medical Science	MOH	District hospital director	Director General for Planning, Health Financing & Information System, MOH	Had multiple opportunities to practice in current role	Stayed due to recognition, promotion, challenging assignments, job satisfaction
P57	Medical Science	MOH	District hospital director	Clinical practice, and management of private health facilities	Had multiple opportunities to apply capacity in former and current role	Left due to family issues, lack of administrative support, career change
P58	Medical Science	MOH	Officer	Director of a unit, MOH	Had opportunity to apply capacity in current and former role	Stayed due to recognition, promotion, challenging assignments
P59	Medical Science	MOH	Researcher and programme manager	Director of programme, MOH	Had multiple opportunities to practice in current role	Stayed due to recognition by employer, promotion

Most graduates reported that they were able to apply their learning in the fields of management, research, training/teaching, policy development, and training material development. There was, however, diversity in the graduates’ ability, capacity and opportunity to apply newly developed skills and expertise. Clusters of factors across multiple levels emerged in the narratives of participants as influencing the contribution of training to organisational capacity development: relevance of newly acquired knowledge and skills set to role/position, and employer recognition in terms of being promoted to senior posts or being assigned challenging tasks; and motivation and retention of trained staff.

The sections to follow highlight patterns in the data under three interrelated themes of alignment of core competencies and role/position, employer recognition, and retention and turnover.

### Alignment of core competencies and role/position

Fifteen trainees were located in departments at the health ministries or university where human resource management was one of the central foci. Three participants were enrolled in the programme with the understanding that they would transition to a more relevant position during or after the training. They were contract staff working in research projects affiliated with target departments in the universities. Two of them were later integrated into the departments as permanent academic staff with opportunities to teach HR-related courses. However, the other participant because of changes in leadership of the institution, he did not have the same support to enable the transition to a more appropriate position. For the majority of the participants, therefore, their role closely matched their acquired competencies and because they were located in institutions mandated to govern or train health professionals, they could make direct links between competencies and workplace demands.

One of the trainees, P3, took on a series of progressively senior posts in a health ministry, aligned to the competencies acquired in the programme, and this is what he has to say about his experience of applying the competencies.

*[During training] I was working on HR [at the HR department in the health ministry] so whatever we learned … we would practice it. … [We] changed the administration and with the belief that the HR programme is important not just for the Ministry [at national level] but also for every region [sub-national level], we started developing a curriculum and started … the programme [a postgraduate programme in HRM in two local universities]. [P3]*

Another trainee, P35, a lecturer at the university, has integrated his learning from the programme into his teaching and consultancy

*I use all of them [training module materials] because [they] are related to the [subjects] I teach … . The [health workforce development module] … has very good examples of things that we can use simply in our context … . [Explaining further what enabled application of learning] I think it is especially because I am in an environment that I not only have to teach but I have to research, I end up using all the modules. I am also part of the [national] human resources observatory. Surely, I have been invited to be part of this because I am studying human resources development. [P35]*

### Employer recognition

In addition to the relevance of the roles, recognition by the employer in the form of promotion or allocation of challenging assignments were found to enable capacity application.

Seniority was found to afford participants the opportunity to take on more challenging responsibilities, and space to implement their learning. A few of the participants held senior HR-related posts prior to being enrolled in the programme. One of these trainees, P14, was the head of HR in the health department at sub-national level. Informed by his learning and enabled by his senior position and associated network, he accomplished significant changes by decentralising HR departments, creating posts for HR managers at district level, ensuring qualified managers are recruited, and existing staff undergo training in newly initiated HR programmes in a local university.

*[The] experience [from the programme] has increased my capacity a great deal. The project [restructuring] coincided with [my participation in] the HRD MPH. I have used my knowledge [from the programme] to make the most of this [restructuring] project. … You need vision to do anything. … While studying in the programme, I was able to realise all the gaps and weaknesses in the way we are doing things. HRH [department] is full of people who are transferred because of disciplinary reasons [and] who don’t understand the work. It was [considered as] a way of punishment. Until recently HR was not a place for professionals. … [The restructuring] is one of the success stories of HRH programme in [the country]. [P14]*

The narratives show that the majority of participants advanced to progressively more senior positions upon joining the programme, either during the course of the programme or after its completion. This ranged from being officers (human resource/medical/research) to becoming senior leaders at sub-national or national levels in health ministries (cases in point are P3, P8, P15, P58, P59, P56, P37).

Training participants’ promotions to senior posts were reported to be recognition of their qualifications, core competencies, or improved contribution to their departments. One of the trainees, P58, was working as a health officer when she joined the programme. Her response shows that she was promoted to senior leadership position due to her improved contribution to the institution, which in turn opened further opportunities to contribute.

*The division within which I was … it was somehow new. … I was the first person to get an opportunity to study. … [The training] programme equipped me with knowledge on how I can train health workers, [how] I can support them … . [Preparing] the strategic plan … I had to plan activities related to training, supervision, mentorship … all that. … [The modules] related to HRH development, planning and so on contributed to making me confident in my position [as a Director]. … I was like a pillar in my division. Because others were not [skilled enough] … I contributed a lot. I think that is why I was appointed as a Director. [P58]*

P56 was a medical officer in a rural district hospital when he joined the programme. He then became director of the district hospital, and later he was promoted to a senior post at the health ministry. His response illuminates the mutually reinforcing relationship between recognition and application of competencies.

*[W] hen I took [the directorship] position at the rural hospital … my predecessor was not very present … . I started establishing some mechanism of meeting people [regularly] … and involving them to propose solution … . I saw some changes in the way they manage. … I do have materials [from the training programme] on my computer and some books. … It helped me a lot … . I am doing [preparing] some procedure manual [focusing on integrating supportive supervision for the ministry] … I am leading development of [HR] policy for the whole [health] sector … I have been … engaging in leading the process of determining the staffing in health … . [and] leading the process of the HR sustainability … [and] the national health sector research agenda. [P56]*

While such timeous recognition and creation of opportunities generated motivation, delayed employer recognition or lack of communication to this end reportedly led to frustration and lack of motivation in other cases. One of the trainees, P15, was an HR officer in a health department at sub-national level, and he was left frustrated by the absence of mobility to a more senior post or better financial package upon graduation. He expressed his annoyance:

*… . That [lack of recognition or promotion] is disappointing. … . You can build their [staff] capacity, but after building capacity [if] you don’t give them anything ... that is ... inexplicable. [P15]*

It took a while before this trainee got promoted to a position of head of HR in the provincial health department. Protracted administrative processes and poor communication led to turnover in the case of another participant, P57.

### Retention and turnover

Thirteen of the participants stayed in their respective institution after graduation. However, five graduates across the countries left their institution to join international agencies, the private sector, or change career paths. An examination of the motives of participants reveals that a diverse set of intrinsic and extrinsic push and pull factors contributed to their decision to stay or leave: financial incentive, employer recognition (promotion or assignment of challenging responsibilities), career prospect, job security, family circumstances, external job holding opportunities, and change in career path.

Four of the five participants (P8, P14, P39, and P57) who left their home institution had a clinical background. Two of them pursued clinical practice/study; the other two occupied management positions in international agencies.

The narratives of two of the training participants, P35 and P36, one based in university and another in a health ministry, illustrate the diversity of experiences and decisions regarding retention. The university-based trainee, P35, who was a junior lecturer, took on HR-focused teaching and consultancy responsibilities related to his core competency in HRH after enrolment in the training programme. He expressed disappointment at his inadequate salary, but he stated his resolve to stay in the institution.

*I have to do consultancy to have [more] money … to take care of the family. It [consultancy] ends up [being a] big burden because I have to work, work, work. … I am still not thinking of leaving this public sector even though I am not satisfied with the salary because I still want to improve my academic career. [P35]*

The trainee based in a health ministry, P36, was working as a junior manager in a public hospital when he joined the programme. He soon left the health ministry to join an international agency and, later, a private company. He cited dissatisfaction with his salary and career prospects as the main reasons for leaving.

*I [left] to work for [an international agency] as a national HR manager [overseeing] administration and HR issues of 500 employees. After a year, I left the [international agency] to join a private company [as a head of the HR department] because I like to grow and get better opportunity to practice HR. I moved from ministry of health [to international agency] because of money. The pay [at MOH] was very, very low. My net pay at the NGO was double what I earned at ministry of health. When I moved [to the private company], it was almost double what I earned at the [international agency]. [P36]*

The present analysis shows that while role relevance and seniority were enablers of capacity application, a closer look at the differences between the groups who left and those who stayed reveals no clear pattern based on role/position, or recognition (be it seniority or assignment of interesting tasks). There were also no country-specific patterns.

## Discussion

The programme discussed in this paper set out to strengthen leadership and training capacity for health workforce development in a regional (African) initiative, working with a small group of locally chosen students who, it was hoped and assumed, would act as capacity catalysts in their organisations. The findings show how different factors related to training (relevance and appropriateness), trainees (role, seniority, motivation) and organisation (employer recognition, incentive arrangements), and broader context (marketability, and other pull factors) interacted in different ways to generate very diverse outcomes of training to organisational capacity development.

The literature also emphasises the dynamic and context dependent nature of capacity development and that it operates not just at individual level but also within the organisations in which individuals operate and the broader environment in which the organisations are located [9, 18–24]. Baser and Morgan (2008) speak about capacity emerging out of ‘a complex interplay of attitudes, assets, resources, strategies and skills, both tangible and intangible (9). Similar views about complexity of capacity development interventions are echoed in the literature in the context of human resources for health (HRH) [7, 25, 27, 28].

All participants spoke about the relevance of learning and the appropriateness of the curriculum and 17 participants found ways to apply the knowledge and skills acquired in their home institutions. While eight participants pursued new opportunities within their ministries, within and beyond HR directorates, three participants based in academic institutions began to incorporate teaching about HRH into the curriculum. For five participants, however, the programme became a catalyst to pursue their careers elsewhere, two in the private sector, two others in similar fields within international agencies. Four of the five who left their home institutions had a prior clinical background. The findings suggest greater turnover propensity and labour market demand for graduates with a prior clinical background. Financial incentives and opportunities presented in private and international agencies played an important role in their decision.

The diversity of capacity application experiences among participants ranges from ones where contribution is optimal to those where contribution remains latent or minimal. It was evident that the translation of learning into organisational contributions was enabled by the alignment between the core competencies of the training and the profile of recruited candidates who either had HR-related responsibilities (training or leadership) or were expected to move into such positions post training. Target institutions (health ministries and local universities) involvement in student selection undoubtedly assisted the alignment of potential to apply new competencies with existing or future roles and functions. The body of literature on training initiatives in general – and leadership development in particular – underscores the importance of ensuring that trainees have the opportunity to utilise newly developed skills and expertise in their local contexts [21, 51, 52], and that this be already taken into account when selecting candidates for training [21, 53].

Beyond competency, the findings suggest that capacity application is influenced by seniority, recognition and support from an employing institution, or application and motivation of participants. The relational aspect of capacity application is further highlighted in the literature as one of the factors facilitating the success of change agents in integrating innovations in their context [32], which most of these graduates demonstrate as they took on the responsibility of spearheading changes in HRH practice in their context. The more senior they were, the more support/resources they had at their disposal and the better positioned they were to consolidate the changes. The above findings also reinforce the significant influence the employing organisation has in determining application by either availing opportunities or support to embed learning in practice.

Motivation (through either intrinsic or extrinsic incentives) seems to influence the likelihood of trainees identifying and capitalising on opportunities to apply learning in their home institutions. As depicted in the experiences of five of the cases, participants may choose to leave the home institution for private or international agencies to advance their career or for better remuneration. In contrast, most participants stayed in the institution, mainly because of job satisfaction or opportunities to advance their career, despite being unhappy with their salary. In the case of academic institutions, the holding of multiple jobs ameliorated the tension around salaries.

Retention represents one of the issues that accounts for the complexity of the link between individual capacity development through training and the improved capacity of organisations in terms of improved performance. A systematic review of the motivation and retention of health workers in developing countries [54] emphasised the importance of recognition in health worker motivation, and identified a set of influential factors, namely, ‘financial incentives, career development and management issues’ [54]. The study showed that trainees exercise their agency to resolve dissonance or reinforce alignment between their best interests and that of the institution. This was evident in this study through the decisions of trainees. Individuals in comparable situations/positions/background, end up making varying decisions as to whether they stay or leave, due to the multiple intersecting factors. In three cases participants had the opportunity to augment their income through their engagement in external multiple job holding practices, thus keeping them in their posts but creating other consequences impacting on organisational capacity [25, 55, 56].

The narratives further show that while participation in the training was reported to improve staff contribution in most instances, the study suggests that training also has the unintended consequence of increasing turnover due to the marketability of newly acquired competencies and leads to capacity loss, as identified in several studies [55, 57, 58]. An exploratory study in four eastern African countries focusing on competency gaps in health workforce management found, for example, that the health ministries of these countries fared poorly compared to the private and non-profit sectors in putting retention strategies in place [59]. These studies have resonance with our findings that the target institutions, which happen to be part of the public sector, stand to lose their trained professionals as they continue to fare unfavourably with private and international agencies.

Another influential individual level factor was seniority, relevant both as an incentive for retention and advantage for the application of learning. It was evident in the accounts of some of the cases that participants in senior positions had better opportunities to take on challenging assignments, with much wider implications, such as the development of HR units, the structuring of HR departments, the training and staffing of these departments, and representing their institutions in specialised networks and partnerships. It is safe to suggest that their seniority gave them the edge (power, decision space, or opportunity) to apply their learning on a national or regional level, or the incentive to remain in the institution despite their relatively low salary. In contrast, three training participants who retained relatively junior positions reported that they were frustrated with the lack of recognition/promotion or opportunity.

Albert Hirschman’s Exit, Voice and Loyalty Framework [60, 61] helps shed light in understanding trained staff’s decisions, as it recognises the heterogeneous ways individuals respond to unfavourable circumstances in an organisation: exit (leave the organisation), voice (express their dissatisfaction and seek change), or remain loyal (quietly put up with the situation). According to this perspective, “exit and voice are the two main responses to dissatisfaction, with voice being more effective and desirable. … a lack of exit opportunities increases voice, and loyalty reduces exit” [60]. In the context of this study, all trainees were based in public institutions, which are generally characterised by low wages and poor working conditions. Our analysis shows that while five chose to leave the institutions (exit), 13 remained (voice/loyal). Our study suggests that, in addition to wages and working conditions, factors related to employer recognition of trained staff (promotion/opportunities) and career prospect inform the choice of trained staff to exit or not. While the situation of those who exit the group is relatively clear, with the limited data we have it is difficult to differentiate between the voice and loyal responses. Hence, a full application of Hirschman’s framework with additional data may generate more nuanced insight about responses of trained staff.

**Fig. 2:**
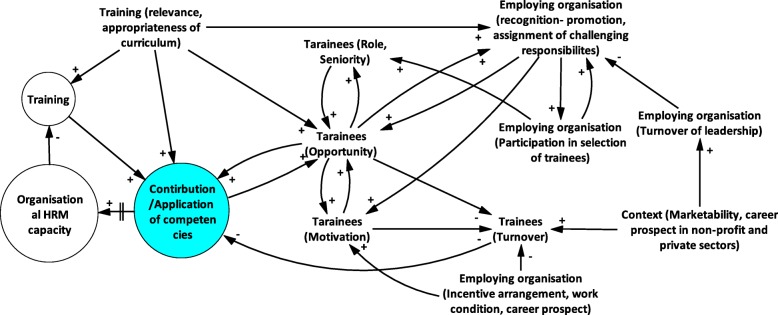
Causal loop diagram of factors mediating contribution of training to organisational capacity development

Figure 2 illustrates the influence that the above discussed contextual and relational factors across multiple levels have on the contribution of training to organisational capacity development – a conclusion, which is supported by the literature [7, 27, 28]. Capacity gaps in HRM lead to training interventions, which equip participants with relevant skills and knowledge. Application of acquired competencies or the resulting improvement in quality and performance leads to gradual reduction of the organisational capacity gap in HRM. This balancing loop is subject to influence by other intersecting factors that particularly influence application of competencies by training participants. Specifically, application/contribution is a function of trainees’ motivation and opportunities to apply learning. The more motivated they are, or the more opportunities at their disposal, the more likely they can contribute. Figure 2 also highlights the various factors related to the employing organisation (recognition via promotion/assignment of relevant tasks, institutional incentive arrangements) influencing motivation and opportunities. Contextual factors (such as labour market or public sector policies) influence the aforementioned organisational level determinants. Each training participant experience unique intersections of these factors and depending on perception of their circumstances, training participants decide to stay and contribute to varying degrees, or leave the institution altogether. In the event of turnover of trained personnel, the HRM capacity shortages in the organisation persist.

Recall and social desirability bias are possible limitations of the study. We strove to address such bias through long-term engagement, building trust and confidence to enable opportunities for open reflection and learning [50]. Another limitation of the study pertains to the fact that dealing with issues related to impact and sustainability, which are key but long-term aspects of capacity development, was not feasible within the limited period of this research. Hence, the research was limited to investigating processes, and short and medium-term outcomes of capacity development.

The findings of the study are not generalizable to participants of other training programmes due to the qualitative research design and the use of purposive sampling and small sample size. A future comparative research examining similar in-service trainings and their relative contribution can help advance understanding and inform policy and practice.

## Conclusion

The present case study highlights the complex contextual and relational factors that affect the contribution of training to organisational development. The results show diversity in graduates’ ability, capacity and opportunity to apply newly developed skills and expertise. The paper argues that training, even if relevant and applicable, makes a ‘latent’ contribution which is activated and realised (or not) through the interaction of multilevel and interacting contextual and relational factors. The study clearly shows how a divergence in individual and organisational goals and expectations (related to financial incentives, work conditions or career path) leads to internal or external migration of trained personnel, which drains an institution of its capacity. Dissatisfaction with payment coupled with lack of opportunity to advance career and marketability of new qualification have led to turnover of trained personnel. The study further implies that implementers need to focus more deliberately on the likely interaction and best possible alignments between training relevance, student selection for potential to contribute, recognition and career advancement potential.

## Data Availability

The data that support the findings of this study are available on request from the corresponding author. Due to the small number of research participants, public availability of the data could compromise research participant privacy and consent.

## Abbreviations

HR: Human Resource
HRH: Human Resources for Health
HRM: Human Resource Management
MOH: Ministry of Health
NGO: Non-governmental Organisations
UWC: University of the Western Cape
WHO: World Health Organisation
